# High Precision, Small Size and Flexible FBG Strain Sensor for Slope Model Monitoring

**DOI:** 10.3390/s19122716

**Published:** 2019-06-17

**Authors:** Hongbin Xu, Xinyu Zheng, Weigang Zhao, Xu Sun, Feng Li, Yanliang Du, Bo Liu, Yang Gao

**Affiliations:** 1 School of Civil Engineering, Beijing Jiaotong University, Beijing 100044, China; 2 Structural Health Monitoring and Control Institute, Shijiazhuang Tiedao University, Shijiazhuang 050043, China; zhengxinyu_sjz@163.com (X.Z.); zhaoweig2002@163.com (W.Z.); du_yanliang@163.com (Y.D.); gaoyang_sjz@163.com (Y.G.); 3 Geotechnical and Structural Engineering Research Center, Shandong University, Jinan 250061, China; 4 Key Laboratory of Structural Health Monitoring and Control, Hebei Province, Shijiazhuang 050043, China; 5 School of Civil Engineering, Southwest Jiaotong University, Chengdu 610031, China; 15690322103@163.com; 6 School of Civil Engineering, Wuhan University, Wuhan 430072, China; lf260986@semi.ac.cn

**Keywords:** fiber Bragg grating, FBG strain sensor, slope model, monitoring, landslide

## Abstract

In this paper, a soft fiber Bragg grating (FBG) strain sensor was constructed of a rubber strip, FBGs and steel plates, which exhibits the advantages of high precision and a small size. A series of FBGs was uniformly pasted on a flexible rubber strip which can monitor the slope deformation by measuring the bending deformation of the rubber strip. Most notably, this sensor can be used to monitor horizontal displacement in the subsurface of the slope model. The relationships among the bending angle of the rubber strip, the strain of the rubber strip, and the subsurface deformation of the slope model were established. In addition, the subsurface deformation of the slope model can be obtained by the FBG strain sensor monitoring. Since a rigid-flexible structure was formed by uniformly pasting a series of steel plates on the other side of the rubber strip, the sensitivity of the FBG strain sensor was improved to be 1.5425 nm/°. The measurement results verify that the FBG strain sensor shows good performance, and the model test results demonstrate that the FBG strain sensor can be used for monitoring the subsurface deformation of the slope model.

## 1. Introduction

Landslides have attracted great attention, as they are serious geological hazards that cause a significant loss of property and human lives. Thus, it is important to study the slippage mechanism of the high and steep slopes and then forecast the landslide [1].

The slope model test is an essential means to study the slip mechanism of the slope, and the corresponding deformation monitoring is necessary [2]. However, the common tilt sensor with a large size is not suitable for monitoring slope model deformation. Generally, traditional slope model monitoring technologies include a multi-point displacement meter (MPDM) [3], non-contact remote laser Vibrometry, calipers [4], photograph measure and CCD laser displacement sensor [5]. Most of the technologies are used to monitor the surface deformation of the slope model, while MPDM technology can monitor the subsurface deformation of the slope model. However, since the fixed end of the MPDM is difficult to be combined with soil firmly, the deformation of the slope model is unavailable to be monitored accurately.

The fiber optic sensing technology is characterized by strong anti-electromagnetic interference [6], strong corrosion resistance, high resolution and long-distance transmission [7]. It has been widely applied in many fields of the slope, such as the monitoring of surface deformation [8] and subsurface deformation [9] of the slope, the strain field [10,11] and the soil pressure [12] of the slope, the hydraulic characteristics [13,14] and the rockfall [15]. In the slope model test, fiber optic sensing technology has been used to monitor surface deformation [16] and subsurface strain [17] of the slope model, and multi-parameter monitoring for the geotechnical centrifuge model [18,19]. However, few studies on the fibre optic sensing were conducted to monitor the subsurface deformation of the slope model.

In this study, a high precision, small size and flexible FBG strain sensor was made to monitor distributed horizontal displacement in the subsurface of the slope model. A series of FBGs was uniformly pasted on a flexible rubber strip to measure the bending deformation of the rubber strip. The deformation of the rubber strip is considered to be the slope deformation. Moreover, a series of steel plates on the rubber strip is beneficial to form a rigid-flexible structure, which improves the sensitivity of the FBG strain sensor to be 1.5425 nm/°. Meanwhile, the relationships among the bending angle of the rubber strip, the strain of the rubber strip, and the subsurface deformation of the slope model were established. Finally, the subsurface deformation of the slope model was measured by the FBG strain sensor.

## 2. Design and Fabrication of the FBG Strain Sensor

**Figure 1:**
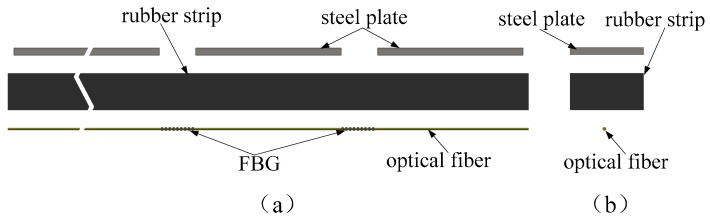
Exploded view of the FBG strain sensor. (**a**) the front view, (**b**) the side view.

**Figure 2:**
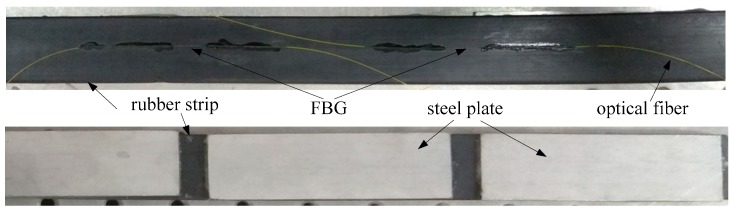
The photo of the FBG strain sensor.

Figure 1 presents the exploded view of the developed FBG strain sensor, which is composed of a rubber strip, steel plates and FBGs. The rubber strip had the best overall performance combination and adequately high elongation capability, with a small stiffness and good fatigue life. Meanwhile the steel plate was characterized by large stiffness and small deformation, and the FBG was characterized by high strain sensitivity. In the fabrication process of the FBG strain sensor, a series of steel plates was uniformly pasted on the one side of the rubber strip, while a series of FBGs was uniformly pasted on the other side of the rubber strip. The FBGs were pasted on a section of rubber strip without pasted steel plate, as shown in Figure 2. When the FBG strain sensor bent, the deformation of the FBG strain sensor occurred on the section without steel plate due to its large stiffness.

## 3. Analysis of the FBG Strain Sensor

**Figure 3:**
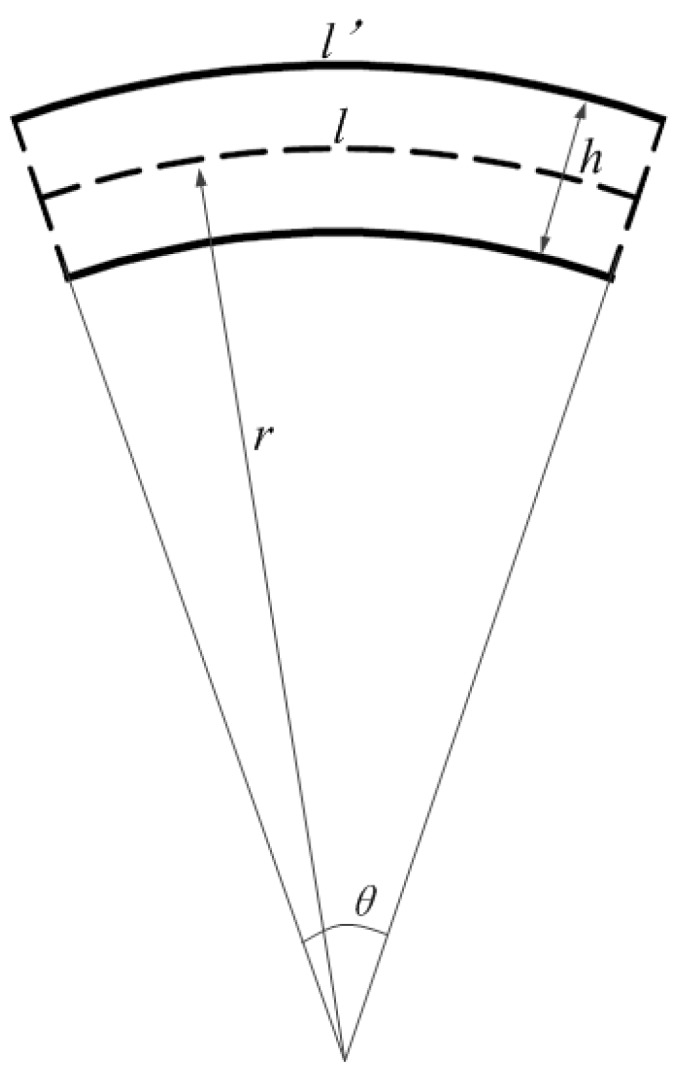
The schematic of the FBG strain sensor under pure bending.

The structure of the developed FBG strain sensor was designed based on the mechanism of beam bending [20]. It is known that the convex surface of the rubber strip is in tension when the rubber strip is bending. After the linear strain of the convex surfaces is obtained, the bending curvature of the rubber strip can be inferred by the beam theory. Since the strain is related to the change of the center wavelength of the FBG, the bending angle of the rubber strip can be measured. The schematic of the FBG strain sensor under pure bending is shown in Figure 3.

Based on the geometry theory, the relationship between the length and angle is expressed by

(1) l=rθ

(2) $$l\prime =(r+\frac{h}{2})\theta$$

where *l* is the length of the section of rubber strip without steel plate, *l*′ is the length of the convex surfaces of the s rubber strip section without steel plate, *r* is the cylinder radius of the rubber strip, *θ* is the bending angle of the rubber strip, and *h* is the thickness of the rubber strip.

From the theoretical mechanics, the strain of the convex surfaces of the rubber strip section without pasted steel plate is expressed by

(3) $$\varepsilon =\frac{l\prime -l}{l}$$

where *ε* is the strain of the convex surfaces of the rubber strip without steel plate.

The pasted FBGs can measure the strain of the convex surfaces of the rubber strip, the relationship between the center wavelength and the strain of the FBG is as follows [21]:

(4) $$\frac{\Delta \lambda }{\lambda }=(1-P_{e})\varepsilon$$

where Δ*λ* is the change of the center wavelength of the FBG caused by the strain *ε*, *λ* is the center wavelength of the FBG, and P_e_ is the elastic-optical coefficient of optical fiber.

The sensitivity of the FBG strain sensor is expressed by

(5) $$S=\frac{\Delta \lambda }{\theta }$$

From Equations (1) to (5), the sensitivity of the FBG strain sensor is expressed by

(6) $$S=\frac{\Delta \lambda }{\theta }=\frac{(1-Pe)\lambda h}{2l}$$

**Figure 4:**
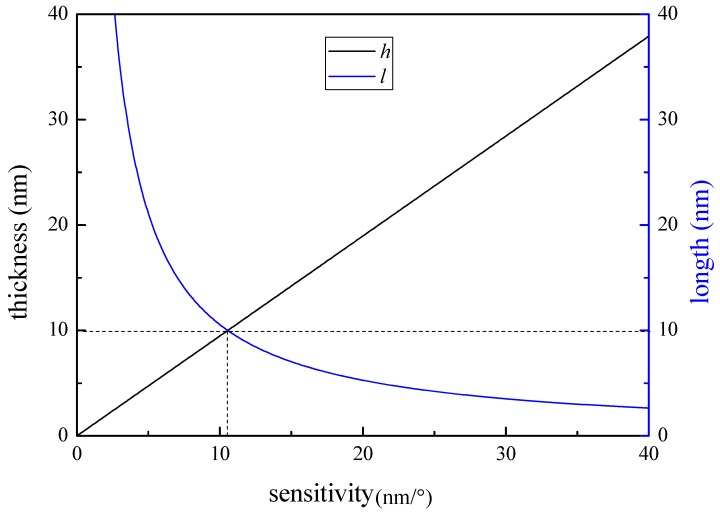
The relationship among *S*, *h* and *l*.

According to Equation (6), the relationship among *S*, *h* and *l* is plotted in Figure 4. This figure demonstrates that the sensor sensitivity dramatically increased by reducing the value of *l*, but the value of *l* should not be less than the length of the FBG; the length of the FBG is about 10 mm.

Since the FBG strain sensor is not only subjected to bending under confinement by steel plates, a correction factor is introduced to Equation (6)

(7) $$S=\frac{\Delta \lambda }{\theta }=K\frac{(1-Pe)\lambda h}{2l}$$

## 4. Measurements and Calculation of the FBG Strain Sensor

**Figure 5:**
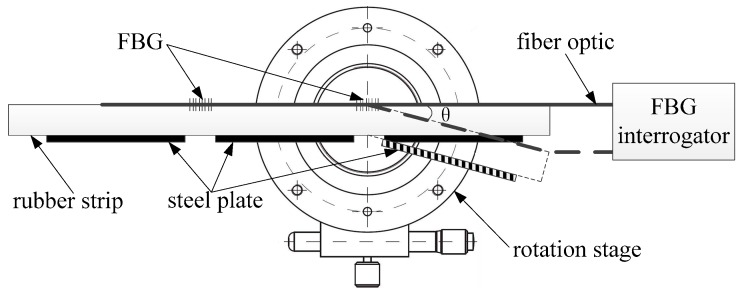
The test system of the FBG strain sensor.

The FBG strain sensor was calibrated on a rotation stage (KSMR100, produced by Zolix, China) with an angle measurement range from 0 to 360° with an accuracy of 0.01°. In addition, an FBG interrogator (SM125, produced by MOI, America) with a wavelength resolution of 1 pm was selected. The test system is shown in Figure 5.

**Figure 6:**
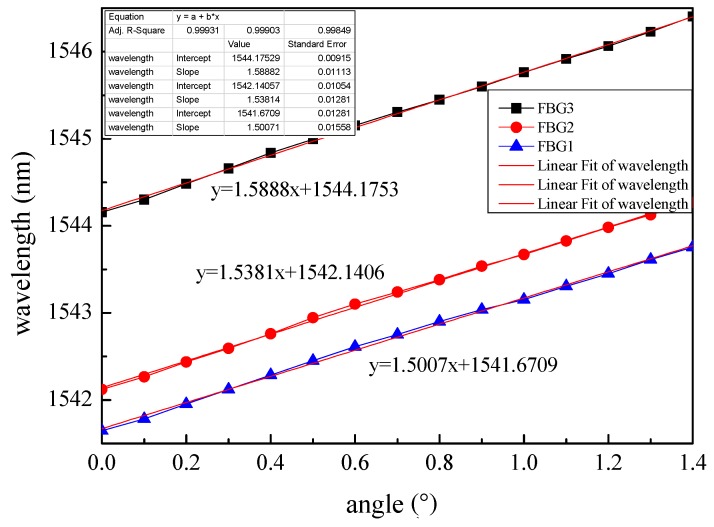
The test result of the FBG strain sensor.

According to Figure 4, when the thickness of the FBG strain sensor was 10 mm, the length of the rubber strip section without pasted steel plate was 10 mm; three parts of the FBG strain sensor were tested. The sensitivities of the FBG strain sensors are 1.5007 nm/°, 1.5381 nm/°, 1.5888nm/° by linear least-squares regression of all measurement points, when the center wavelength of the FBGs are 1541.646 nm, 1542.121 nm, 1544.156 nm, as shown in Figure 6. Therefore, the average sensitivity of the FBG strain sensor was about 1.5425 nm/°. The calculated average value of the K was 0.1469, on the basis of the sensitivity of the FBG strain sensor, size parameters of the rubber strip and Equation (7).

## 5. Slope Model Experiment and Results

### 5.1. Experimental Principles and Methods

**Figure 7:**
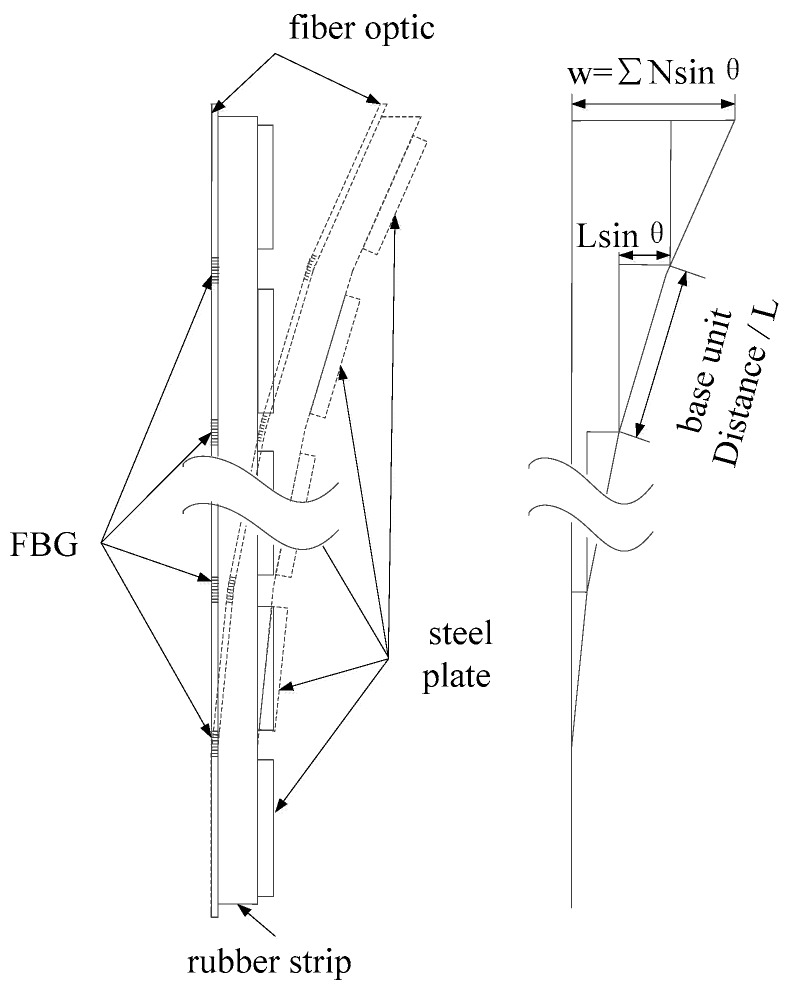
The FBG strain sensor and its measuring principle.

The displacement measurement is of great significance in monitoring landslides. The FBG strain sensor can indirectly measure the displacement of the slope model. When the slope model slides, the installed FBG strain sensor in the slope model will bend as well. The bending angle of each position of the slope model can be calculated by the change of the center wavelength of the FBG, and then the displacement of each position of the slope model can be calculated [22], as shown in Figure 7.

Based on the measuring principle, the relationship between the displacement and angle is expressed by

*w_i_* = *L*sin*θ_i_* ≈ *Lθ_i_* (8)

where, *w_i_* is the displacement of the *i*th unit of the slope model, *L* is the base unit distance of the FBG strain sensor, *i* is the location number of the *L*.

The displacement of the slope model can be described as

(9) $$W=\sum_{i=1}^{n}(n+1-i)w_{i}$$

**Figure 8:**
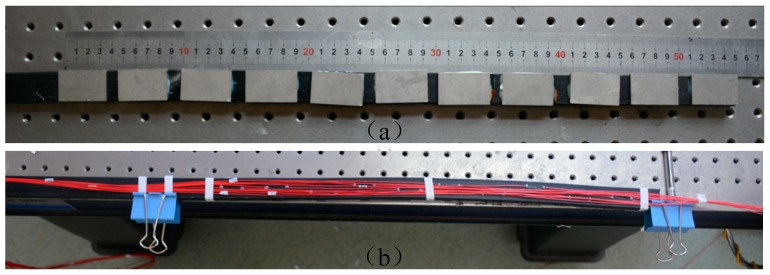
The photo of the FBG strain sensor used in the slope model monitoring. (**a**) the side of the rubber strip pasted with steel plates, (**b**) the side of the rubber strip pasted with FBGs.

According to the above results, an FBG strain sensor with ten FBGs with different center wavelength was prepared, as shown in Figure 8.

### 5.2. Experimental Results and Analysis

**Figure 9:**
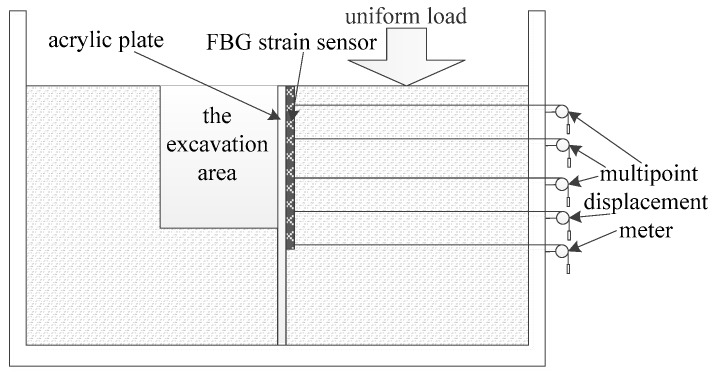
Experiment Scheme of the test model.

A model test was carried out to verify the performance of the FBG strain sensor by digging the backfill soil in a steel structure frame, as shown in Figure 9. The size of the test model was 3 m long, 1 m wide and 1.2 m high, while the excavation area was 0.4 m width and 0.6 m away from the left edge of the test model. To increase the deformation of the model, a uniform load of 14 kN was applied on the right side of the excavation area. A series of MPDMs was equipped to monitor its deformation during the excavation process in the model test. Meanwhile, an acrylic plate was set at the excavation boundary to install the sensor conveniently, and one end of the MPDM and the FBG strain sensor were installed on the acrylic plate.

**Figure 10:**
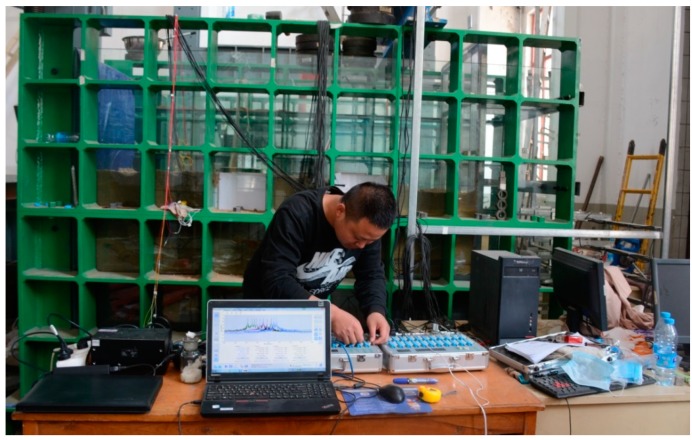
Photo of the test model.

**Figure 11:**
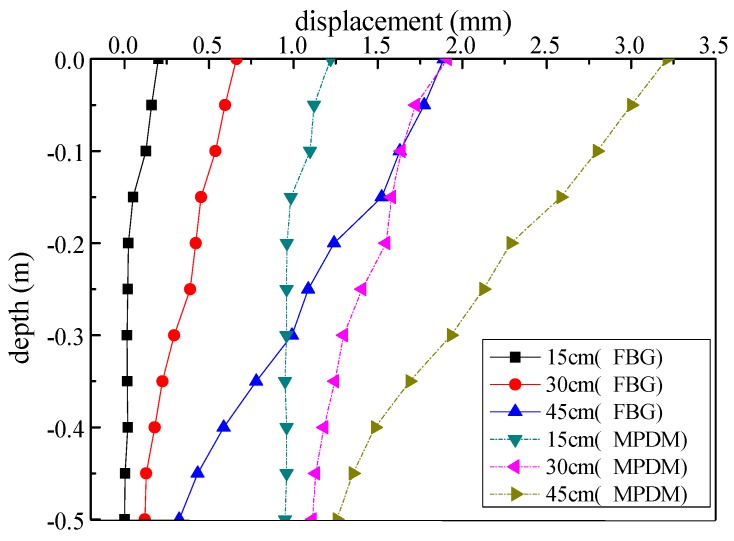
The test results of two monitoring methods.

**Figure 12:**
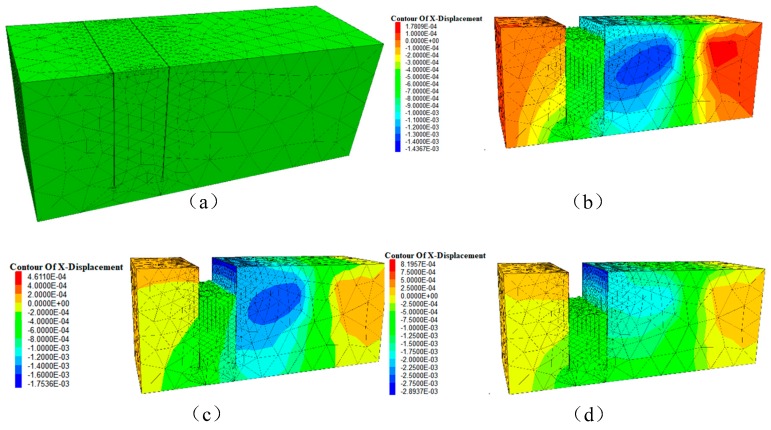
The displacement analysis of the test model. (**a**) finite element model, (**b**) displacement nephograms of the test model with an excavation depth of 15 cm, (**c**) displacement nephograms of the test model with an excavation depth of 30 cm, (**d**) displacement nephograms of the test model with an excavation depth of 45 cm.

**Figure 13:**
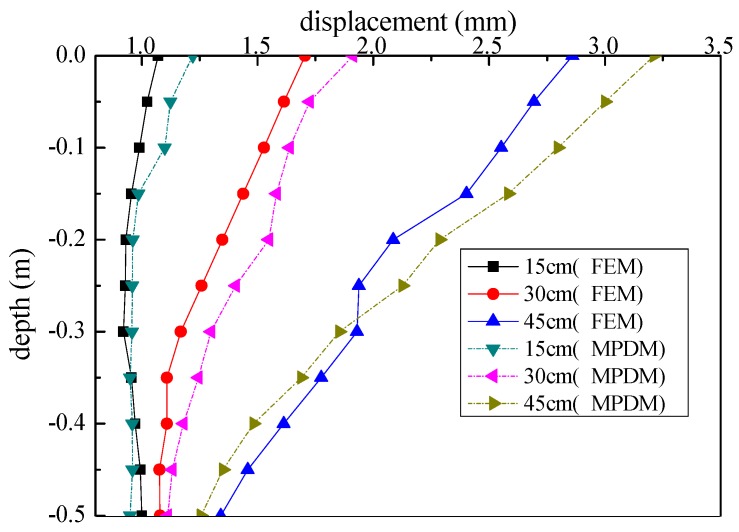
The results of the displacement calculated by the FEM and the results of the MPDM monitored.

The excavation model tests were carried out in three stages with each excavation depth of 15 cm, 30 cm, 45 cm, respectively. The displacement measurements by using these two methods were shown in Figure 10. The obtained results are shown in Figure 11, which presents obvious differences in the monitoring result. A finite element model (FEM) was applied to analyze the deformation of the test model, as shown in Figure 12. Besides, the deformation of the FEM was compared with the MPDM monitoring results. It is found that, the deformation results calculated by the FEM are similar to the MPDM monitoring results, as shown in Figure 13.

**Figure 14:**
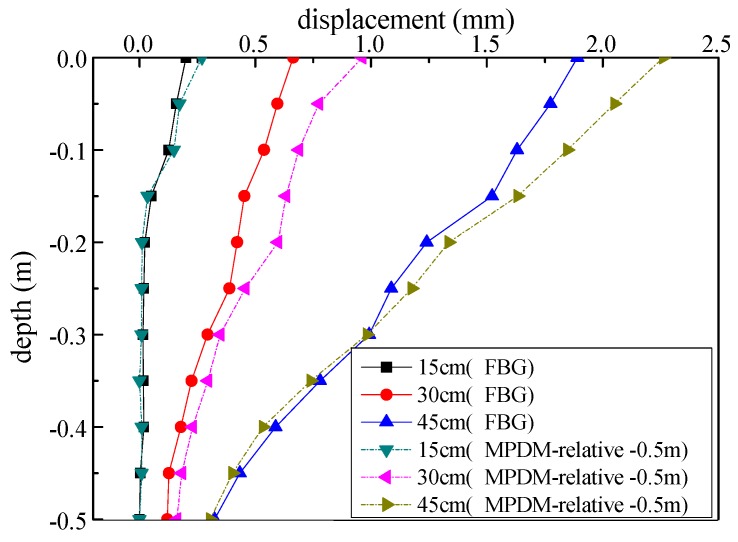
The result comparison of two monitoring methods.

Compared to the results of the finite element analysis and the MPDM, the excavation boundary of the test model with sensors slipped as a whole body under external load, while the displacements were different at different positions. The testing principle of the FBG strain sensor is to measure the relative displacement and inability to monitor the displacement of the test model with undifferentiated of all parts. Therefore, the results of the FBG strain sensor were smaller than those of MPDM. The results of FBG strain sensor were similar to the relative monitoring results of MPDM relative to itself −0.5 m, as shown in Figure 14.

## 6. Conclusions

In this study, an FBG strain sensor was developed to monitor the displacement of the slope model by measuring the strain of the bending rubber strip. The design, fabrication, and structural analysis of the FBG strain sensor were introduced in this paper, respectively.

The test results confirmed the feasibility of the FBG strain sensor was verified by successfully monitoring the real-time curvature with a high sensitivity of 1.5425 nm/°.

Both the results of finite element analysis and the MPDM show that the FBG strain sensor can well characterize the slip mode, but cannot detect the overall movement of the model. In addition, the FBG strain sensor should be long enough to determine the slip of the slope model accurately.

As the FBG strain sensor is easily damaged by pulling, the installation should be conducted carefully, and protection measures should also be considered, especially in the next step of the research project.

Since the model experiment took a short time, the temperature of the slope model slightly changed. Thus, the effect of temperature on the FBGs can be ignored, and the temperature compensation was not considered. Therefore, the FBG strain sensor can be widely applied in various fields, with the advantages of low cost, easy fabrication and installation, and excellent performance.
